# M-CSF Induces Monocyte Survival by Activating NF-κB p65 Phosphorylation at Ser276 via Protein Kinase C

**DOI:** 10.1371/journal.pone.0028081

**Published:** 2011-12-22

**Authors:** Yijie Wang, Xiaokui Mo, Melissa G. Piper, Hongmei Wang, Narasimham L. Parinandi, Denis Guttridge, Clay B. Marsh

**Affiliations:** 1 Department of Internal Medicine, The Ohio State University, Columbus, Ohio, United States of America; 2 Department of Human Cancer Genetics, The Ohio State University, Columbus, Ohio, United States of America; Texas A&M University, United States of America

## Abstract

Macrophage colony-stimulating factor (M-CSF) promotes mononuclear phagocyte survival and proliferation. The transcription factor Nuclear Factor-kappaB (NF-κB) is a key regulator of genes involved in M-CSF-induced mononuclear phagocyte survival and this study focused at identifying the mechanism of NF-κB transcriptional activation. Here, we demonstrate that M-CSF stimulated NF-κB transcriptional activity in human monocyte-derived macrophages (MDMs) and the murine macrophage cell line RAW 264.7. The general protein kinase C (PKC) inhibitor Ro-31-8220, the conventional PKCα/β inhibitor Gö-6976, overexpression of dominant negative PKCα constructs and PKCα siRNA reduced NF-κB activity in response to M-CSF. Interestingly, Ro-31-8220 reduced Ser276 phosphorylation of NF-κBp65 leading to decreased M-CSF-induced monocyte survival. In this report, we identify conventional PKCs, including PKCα as important upstream kinases for M-CSF-induced NF-κB transcriptional activation, NF-κB-regulated gene expression, NF-κB p65 Ser276 phosphorylation, and macrophage survival. Lastly, we find that NF-κB p65 Ser276 plays an important role in basal and M-CSF-stimulated NF-κB activation in human mononuclear phagocytes.

## Introduction

Monocytes are produced in the bone marrow and circulate in blood for 24–48 hours [1]. In the absence of serum, monocytes die via apoptosis [1], [2]. Macrophage colony-stimulating factor (M-CSF) stimulates mononuclear phagocyte survival and differentiation [3]. Importantly, M-CSF-mediated cell survival and activation is associated with a variety of human diseases, including atherosclerosis, transplant vascular sclerosis and breast cancer metastasis [4], [5], [6].

We previously identified that NF-κB activation is important in M-CSF-induced monocyte survival [7]. In addition to its role in mononuclear phagocyte survival, the transcription factor NF-κB regulates numerous genes that play important roles in cellular signaling, stress response, cell growth, survival, differentiation and inflammation [8]. There are five members in the NF-κB family: RelA/p65, p50, p52, c-Rel and RelB. The most common activating complex is the p50/p65 heterodimer, driven by the activation domain in the NF-κB p65 subunit. NF-κB p65 regulates important developmental processes [9], [10]. Mice lacking NF-κB p65 die in utero and have extensive liver damage via enhanced apoptosis [9]. Embryonic macrophages from NF-κB p65 null mice are susceptible to TNFα-induced apoptosis which is rescued by overexpressing the NF-κB p65 subunit [10]. Moreover, inhibiting NF-κB induces cell death in many cell types and cytokine-independent survival is mediated by constitutively active NF-κB in murine macrophages [11].

In monocytes and macrophages, NF-κB is an important transcriptional factor for expression of cytokines and cell surface receptors [12]. However, unlike resting T-cells, NF-κB is constitutively present in the nuclei of primary monocytes and monocytic cell lines in the absence of exogenous stimuli as demonstrated by mobility shift analysis [13]. Similarly, constitutively active NF-κB was observed in human alveolar macrophages [14].

In the classic/canonical pathway, the NF-κB p50/p65 complex is sequestered in the cytosol by IκBα [15]. Upon stimulation by cytokines or UV radiation, IκBα is phosphorylated, ubiquitinated, and degraded, releasing NF-κB p50/p65 to translocate to the nucleus and transactivate target genes. After its release from IκBα, NF-κB p65 can undergo post-translational modification to activate gene transcription. The role of NF-κB p65 phosphorylation on NF-κB transcriptional activity varies by stimulus, time of stimulation and cell type [16]. Previous research shows that phosphorylation of NF-κB p65 at Ser276, Ser529 or Ser536 plays an important role in regulating transcriptional activity of NF-κB [17]. In TNFα-treated murine fibroblasts, Ser276 of NF-κB p65 is phosphorylated by MSK1 to enhance NF-κB transcriptional activity [18]. In macrophages treated with endotoxin, NF-κB transcription activity is associated with phosphorylation on Ser276 and Ser536 that is regulated through protein kinase A (PKA) and IKKβ respectively[16], [19]. In human T cells, NF-κB p65 is constitutively phosphorylated on Ser536 to facilitate NF-κB p65 nuclear translocation following cellular stimulation [20]. Accumulating evidence reveals that NF-κB p65 phosphorylation at Ser276 is crucial for its transcriptional activity. Upon nuclear translocation, phosphorylation of Ser276 on NF-κB p65 by PKA recruits the transcription co-activator, p300 to potentiate NF-κB-regulated gene transcription [21]. However, other studies show that PKA inhibits NF-κB-regulated gene expression by stabilizing IκBα [22], [23]. Interestingly, the serine/threonine kinase Pim-1 directly phosphorylates NF-κB p65 at Ser276 by stabilizing to prevent ubiquitin-proteasome degradation [24]. Several other phosphorylation sites are also described to enhance NF-κB gene transactivation [25].

Here, we investigate the role of protein kinase C (PKC) in M-CSF-stimulated NF-κB activation. PKC proteins are multifunctional kinases that differ in structure, function and co-factor requirement [26]. PKCs are involved in diverse cell responses, including cell growth, survival, differentiation and development [27]. The 12 closely related enzymes of the PKC family are divided into three classes: conventional (cPKCs: α βI βII and γ require Ca^2+^ and diacylglycerol (DAG); novel (nPKCs: δ, ε, η, θ and μ) require DAG; and atypical (aPKCs: ξ, ι and λ) require neither Ca^2+^ nor DAG. Monocytes and macrophages predominantly express conventional PKC isoforms (PKCα PKCβI and PKCβII) and novel PKCs (PKCδ and PKCε). Conventional PKCs regulate differentiation of the human promyelocytic leukemia cell line HL60 to macrophages [28]. PKCα induces IL-1α, iNOS and TNFα mRNA production after lipopolysaccharide (LPS) exposure [29]. In addition, accumulating evidence suggests that conventional PKCs like PKCα have anti-apoptotic functions. For example, PKCα is overexpressed in a variety of tumor cells including gliomas, liver, and lung [30], [31]. In epithelial cells, inhibition of PKCα induces PKCδ-dependent apoptosis [31]. Interestingly, in human monocytes and premonocytic THP-1 leukemia cells, novel PKCs like PKCδ have the opposite effect on cell survival, Voss et al showed that PKCδ directly phosphorylates caspase-3 and promotes etoposide-induced apoptosis [32]. Moreover, knockout mouse studies suggest that another novel PKC, PKCε is critically involved in early LPS-mediated signaling in activated macrophages [33].

Previously, we reported that M-CSF promotes monocyte survival through the activation of the PI3-K/AKT pathway [3]. In addition to AKT activation, M-CSF stimulates PKC in human monocytes and increases NF-κB DNA binding [34]. However, whether PKC and/or NF-κB activation is critical in M-CSF-stimulated mononuclear phagocyte survival and/or differentiation is unclear. In other cells, PKC plays an important role in NF-κB activation and cell survival [35], however the specific mechanisms of this activation and the biological effects on cellular phenotype are not known. Therefore, we focused at understanding the role of PKC in the regulation of NF-κB activation and M-CSF-induced monocyte survival.

Here we demonstrates that M-CSF upregulated the NF-κB transcription and cell survival in human and mouse macrophages. This activity was reduced by the conventional, but not novel, PKC inhibitors, dominant negative PKCα constructs or PKCα siRNA. Conventional PKC regulated NF-κB-regulated gene expression and phosphorylation of Ser276 of NF-κB p65 occurred in an M-CSF-dependent fashion correlating with its maximal transcriptional activity. Furthermore, PKCα-regulated phosphorylation of Ser276 of NF-κB p65 plays an important role in regulating its activity in mononuclear phagocytes and murine embryonic fibroblasts.

## Results

### M-CSF Induces NF-κB Transcriptional Activity in Human Monocyte–Derived Macrophages (MDMs) and Mouse Macrophage Cell Line, RAW 264.7

**Figure 1:**
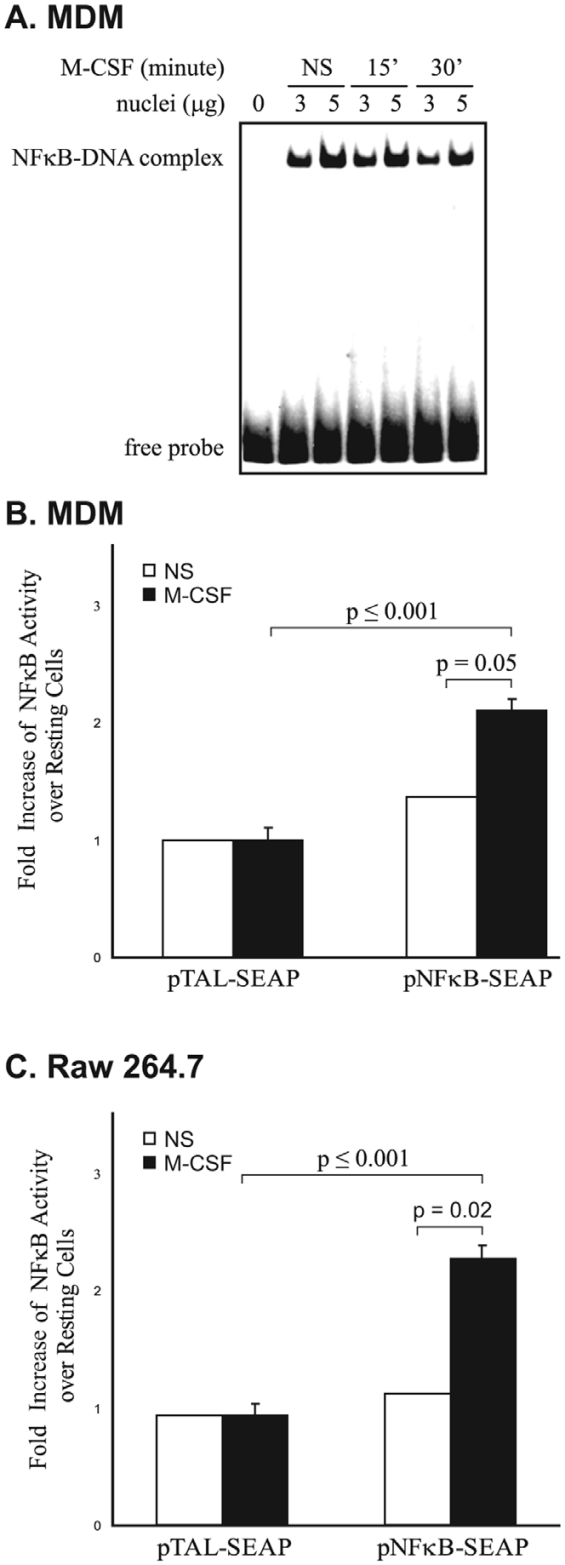
M-CSF induces NF-κB transcriptional activity in macrophages. (**A**) Nuclei were extracted from human MDMs treated without or with M-CSF for 15 or 30 minutes. NF-κB DNA binding activity was analyzed by EMSA Shown is a representative blot from three independent experiments. NS: non-stimulated. (**B**) Human MDMs transiently transfected with pTAL-SEAP or pNF-κB-SEAP constructs were treated with M-CSF in X-vivo medium and incubated for 6 hours before the collection of medium. NF-κB activity was analyzed by measuring the amount of SEAP secreted into the medium and data are expressed as fold increase of SEAP activity over that in pTAL-SEAP transfected resting cells. (**C**) RAW 264.7 cells were transiently transfected with pTAL-SEAP or pNF-κB-SEAP construct. Cells were serum starved for 4 hours prior to 2 hours stimulation with mouse recombinant M-CSF (100 ng/ml). Culture media was collected to measure SEAP production. Data are expressed mean ± S.E.M, for three independent experiments.

To determine if M-CSF induced NF-κB DNA binding in human macrophages, we performed EMSA analysis on nuclear lysates from M-CSF-treated MDMs. Similar to previous reports [11], nuclear NF-κB constitutively bound DNA in non-stimulated monocytes (Figure 1A). Interestingly, adding M-CSF did not alter NF-κB DNA binding by EMSA. In contrast, after transiently transfecting human MDMs with pNF-κB-SEAP constructs containing four NF-κB consensus binding sequences, M-CSF treatment of the transfected cells resulted in a 2.3-fold increase in SEAP release in the culture media compared to PBS (vehicle)-treated transfected MDMs (Figure 1B). As a control, the pTAL-SEAP construct lacking NF-κB binding sites was used. Cells transfected with the pTAL-SEAP construct did not produce SEAP in the absence or presence of M-CSF (Figure 1B).

We next investigated whether M-CSF induced NF-κB activity in the mouse macrophage cell line, RAW 264.7. RAW 264.7 cells were transfected with either the NF-κB-SEAP reporter or control pTAL-SEAP construct. As shown in Figure 1C, M-CSF treatment of RAW 264.7 cells increased NF-κB reporter activity by 2.5-fold over that of non-treated cells. Together, our data demonstrate that M-CSF induced NF-κB transcriptional activity in macrophages.

### PKC Inhibition Reduces NF-κB Activity in Human MDMs and RAW 264.7 Cells

**Figure 2:**
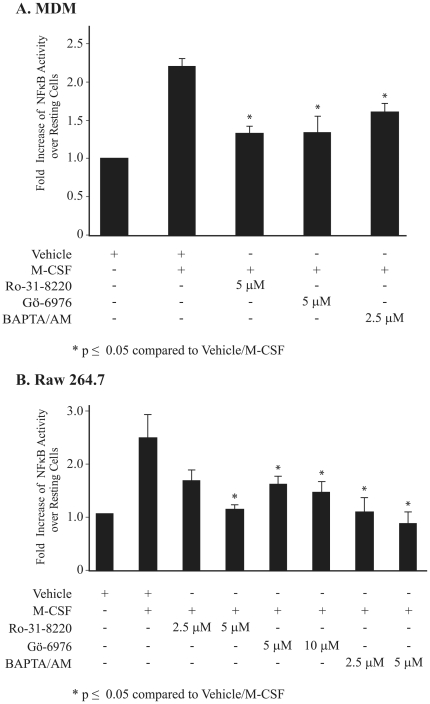
Inhibition of PKC reduces NF-κB activity in M-CSF stimulated macrophages. (**A**) Human MDMs transfected with pNF-κB-SEAP were pre-incubated with inhibitors; Ro-31-8220, Gö-6976, or BAPTA/AM for 30 minutes prior to 6 hours M-CSF stimulation. (**B**) RAW 264.7 cells transfected with the pNF-κB-SEAP construct were pre-incubated with inhibitors for 30 minutes prior to 2 hours of stimulation with mouse recombinant M-CSF. The NF-κB activity was analyzed by measuring SEAP production in the medium and data are expressed as fold increase of SEAP activity over that in pTAL-SEAP transfected resting cells. The graph represents mean ± S.E.M for three independent experiments. *The p-values of cells treated with inhibitors/M-CSF compared to vehicle/M-CSF were ≤0.05.

Since NF-κB is activated by PKC in several cell types [36], we next determined if M-CSF-induced NF-κB transcriptional activity was dependent on PKC activation and if calcium, a co-factor for conventional PKC isoform activation, was important. In addition, because of the number of conventional PKCs existing within mononuclear cells, pharmacological inhibitors of PKC family activation was used to determine the relationship of PKC activation to M-CSF-induced cellular survival. MDMs were transfected with the pNF-κB-SEAP reporter and then treated with the general PKC inhibitor, Ro-31-8220; the conventional PKCα/β inhibitor Gö-6976; or the intracellular calcium blocker BAPTA/AM. Ro-31-8220 significantly suppressed M-CSF-induced NF-κB activity compared to cells treated with M-CSF alone (Figure 2A). In addition, Gö-6976 and BAPTA/AM also blocked NF-κB activity in M-CSF-treated MDMs. Trypan blue exclusion analysis did not indicate cell death suggesting that this suppression was not due to non-specific toxicity. These data indicate that M-CSF mediated NF-κB activation through calcium-dependent conventional PKC activation.

We next investigated whether PKC inhibition affected NF-κB activity in the mouse macrophage cell line, RAW 264.7. Similar to MDMs, PKC inhibitors Ro-31-8220, Gö-6976 and BAPTA/AM reduced M-CSF-induced NF-κB activity in a dose-dependent manner in RAW 264.7 macrophages (Figure 2B). To ensure that PKC specifically regulated NF-κB p65 and not the closely related family member, c-Rel, we co-transfected c-Rel and NF-κB-SEAP constructs into the Raw 264.7 cell line and measured NF-κB activity in response to M-CSF. There was no increased NF-κB activity in cells expressing c-Rel (Figure S1). These observations indicate that PKC functioned upstream of NF-κB p65 in MDMs and RAW 264.7 cells.

### NF-κB and PKC(s) Mediate Human MDM Survival in Response to M-CSF Stimulation

**Figure 3:**
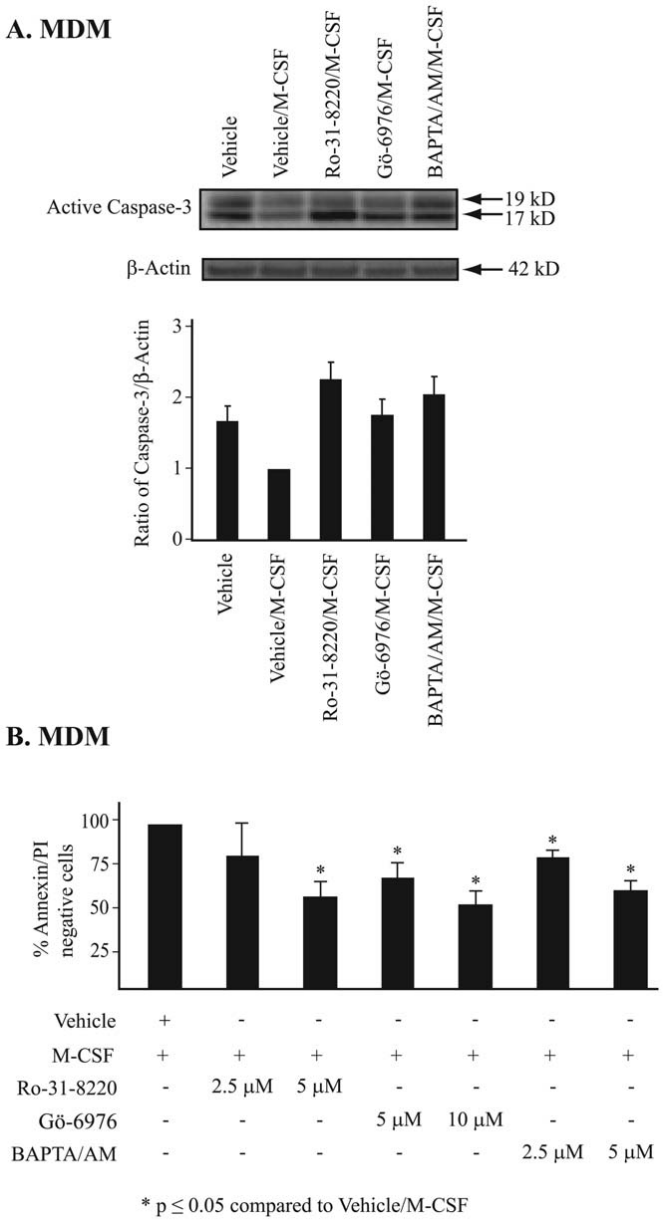
Inhibition of PKC or NF-κB induces apoptosis in MDMs. (**A**) MDMs were pre-incubated in RPMI medium containing inhibitors (Ro-31-8220: 5 µM, Gö-6976: 5 µM, BAPTA/AM: 2.5 µM) for 30 minutes prior to the addition of M-CSF. As a control, untreated cells were incubated with dimethyl sulfoxide (DMSO). Cell lysates were resolved by SDS-PAGE and immunoblotted with antibody recognizing the active cleaved form of caspase-3. The blots were reblotted with β-actin that served as a loading control. The ratio of active caspase-3 bands (17 kD and 19 kD) to β-actin control was determined by densitometry analysis (*bottom panel*). Data represents the mean ± S.E.M from two independent donors. (**B**) Apoptosis of the treated MDMs was also measured by Annexin V-FITC and propidium iodine (PI) staining and analyzed by flow cytometry. The percentage of surviving cells (Annexin V/PI negative) for the cells treated with vehicle/M-CSF was arbitrarily set as 100. Data shown represent the mean ± S.E.M from three independent experiments. *The p-values of inhibitors/M-CSF compared to vehicle/M-CSF were ≤0.05.

Since PKC and NF-κB are critical in cell survival [37], [38], we hypothesized that M-CSF promoted cell survival through PKC in human MDMs. To detect apoptosis, the expression of cleaved caspase-3, a marker of apoptosis, was analyzed in the presence or absence of M-CSF and PKC inhibitors. In MDMs pretreated with PKC inhibitors (Ro-31-8220, Gö-6976, or BAPTA/AM) and stimulated with M-CSF, cleaved caspase-3 was elevated to levels of cells treated with vehicle alone (Figure 3A). In contrast, cells incubated with M-CSF and vehicle had less cleaved caspase-3 than cells incubated with vehicle alone or M-CSF with PKC inhibitors. These data supported the hypothesis that M-CSF-induced NF-κB activity was regulated by conventional PKCs, not novel PKCs.

To confirm that PKC inhibition decreased cell survival, cells were treated with M-CSF in the absence or presence of PKC inhibitors and then also examined for apoptosis by Annexin V/PI staining. PKC inhibitors in the presence of M-CSF reduced the number of Annexin V/PI negative MDMs compared to cells treated with M-CSF alone (Figure 3B). These observations further suggested that MDM survival is promoted by M-CSF and critically involves conventional PKCs and NF-κB activity.

### M-CSF-Induces PKCα Kinase Activity

**Figure 4:**
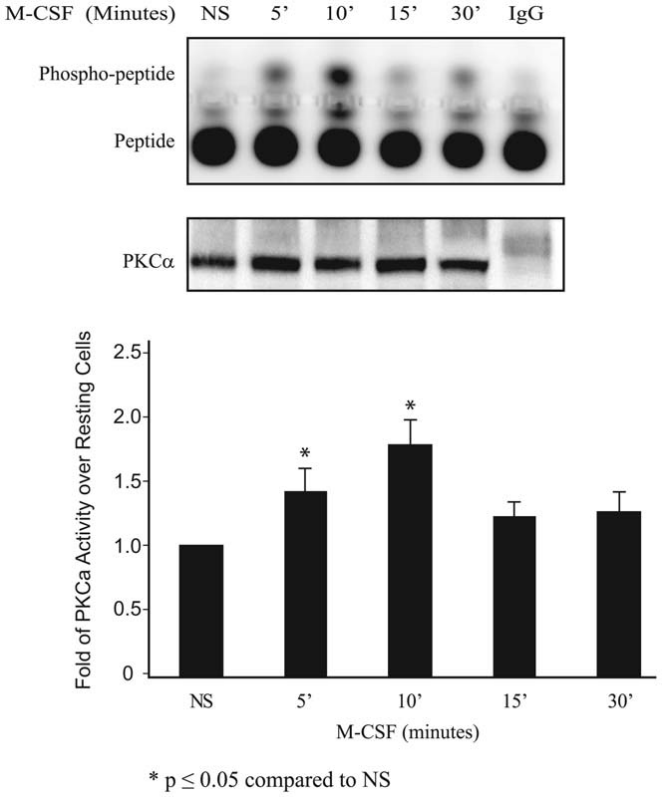
M-CSF activates PKCα in human MDMs. MDMs treated with M-CSF (100 ng/ml) for varying amounts of time were lysed and immunoprecipitated using anti-PKC antibody or control IgG antibody. One half of the samples was used to analyze PKC kinase activity using a fluorescein tagged peptide and visualized by agarose gel electrophoresis (*top panel*), while the other half was subjected to Western blot analysis to confirm equal amounts of PKCα were immunoprecipitated from each sample (*middle panel*). The kinase assay was quantitated using Quantity One software (Bio-Rad) (*bottom panel*). Data represents the average fold increase of PKCα activity in non-stimulated samples compared to M-CSF-treated MDM ± S.E.M for three independent experiments. NS: non-stimulated. * The p-values of M-CSF stimulated compared to non-stimulated were ≤0.05.

Since our data suggested that conventional PKCs were involved in activating NF-κB in response to M-CSF in primary human macrophages, we next investigated whether the conventional PKC, PKCα was a downstream target of M-CSF in human MDMs. PKCα was immunoprecipitated from human MDMs at the indicated time points (Figure 4), and then a PKC kinase assay was performed using a fluorescein-tagged peptide as a substrate. As shown in Figure 4 (upper panel), the peptide substrate was maximally phosphorylated within 10 minutes of stimulation (1.8-fold over resting cells, lower panel), and then returned to basal levels by 15 minutes. This effect was not seen in M-CSF-stimulated monocytes when isogenic antibodies (IgG) were used for immunoprecipitation. Western blots of identical samples using an antibody recognizing PKCα demonstrated that equal amounts of PKCα were assayed (Figure 4, middle panel).

### M-CSF-Dependent Activation of PKC Does Not Regulate the Classical NF-κB p65 Activation Pathway

**Figure 5:**
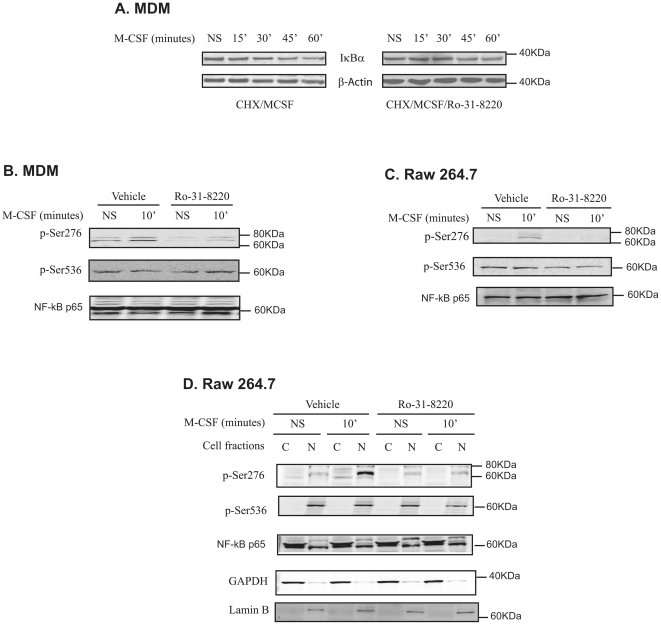
M-CSF-induced PKC activity does not regulate IκBα degradation but regulates the phosphorylation of NF-κB p65 at Ser276. (**A**) MDMs were pretreated with cycloheximide (CHX) in the absence or presence of Ro-31-8220 for 30 minutes prior to M-CSF stimulation for the indicated times. Whole cell lysates were subjected to Western blotting with anti-IκBα antibody. Data are representative of three independent experiments. (**B**) MDMs were pretreated with either vehicle or Ro-31-8220 for 30 minutes before the addition of M-CSF for 10 minutes. Whole cell lysates were resolved by SDS-PAGE and phospho-Ser276 or phospho-Ser536 of NF-κB p65 was detected using phospho-specific antibodies to either residue of NF-κB p65. (**C**) Whole cell lysates of RAW 264.7 cells treated with vehicle control or Ro-31-8220 in the absence or presence of M-CSF were subjected to Western blot analysis with phospho-Ser276 or phospho-Ser536 NF-κB p65 antibodies. (**D**) Cytosolic and nuclear fractions of RAW 264.7 were obtained from the treated cells and immunoblotted for phospho-NF-κB. The purity of the cytosolic and nuclear fractions was confirmed by immunoblotting with GAPDH and Lamin B, respectively. Shown are representative blots from three independent experiments.

Canonical activation of NF-κB occurs via phosphorylation and degradation of IκBα leading to the release and nuclear translocation of the NF-κB p50/p65 heterodimer to transactivate target genes [37], [39]. Since conventional PKC activity was important in regulating M-CSF-induced NF-κB activation, we next investigated whether IκBα degradation was regulated by PKC. Cells were treated with cyclohexamide (CHX) to inhibit protein synthesis of IκBα, and its degradation was followed. As shown in Figure 5A, PKC inhibition with Ro-31-8220 did not alter M-CSF-induced IκBα degradation, suggesting that M-CSF-induced PKC activity augmented NF-κB transcriptional activity by an alternative pathway, like post-translational modification of NF-κB p65.

### M-CSF Induces Phosphorylation of NF-κB Ser276 in a PKC-dependent Fashion

Since M-CSF did not regulate NF-κB activation by influencing IκBα, we next sought to determine if M-CSF affected NF-κB p65 by post-translational mechanisms. Thus, we examined the phosphorylation of NF-κB p65 with specific phospho-NFκB p65 (Ser276 and Ser536) antibodies. M-CSF induced the phosphorylation of Ser276 but not Ser536 of NF-κB p65 in MDMs. Compared to vehicle, the general PKC inhibitor Ro-31-8220 reduced Ser276 phosphorylation, but not Ser536, phosphorylation in M-CSF-stimulated cells (Figure 5B). Furthermore, M-CSF-stimulated NF-κB p65 phosphorylation at residue Ser276 in RAW 264.7 cells was also PKC dependent (Figure 5C). These studies suggested that PKC(s) regulated Ser276 phosphorylation but not Ser536 in both human MDMs and mouse macrophages after M-CSF stimulation.

We next performed cellular fractionation to identify the cellular location of phosphorylated NF-κB p65 in Raw 264.7 cells. Non-phosphorylated NF-κB p65 was located in both cytosolic and nuclear fractions, but phosphorylated Ser276 and Ser536 NF-κB p65 was primarily located in nuclear fraction after M-CSF stimulation (Figure 5D). Notably, constitutive phosphorylation of Ser536 NF-κB p65 was found in these cells. Importantly, Ro-31-8220 reduced M-CSF-induced Ser276 phosphorylation of NF-κB p65 in both the cytosolic and nuclear fractions, while M-CSF-induced NF-κB p65 Ser536 phosphorylation was present in the nucleus regardless of PKC inhibition. These observations indicate that M-CSF-induced Ser276 and Ser536 are regulated differently by conventional PKC activation in mononuclear phagocytes. The purity of the cytosol and nuclear cell fractions was confirmed by immunoblotting with GAPDH and Lamin B, respectively (Figure 5D).

### M-CSF-dependent PKC Regulates NF-κB-targeted Genes

**Figure 6:**
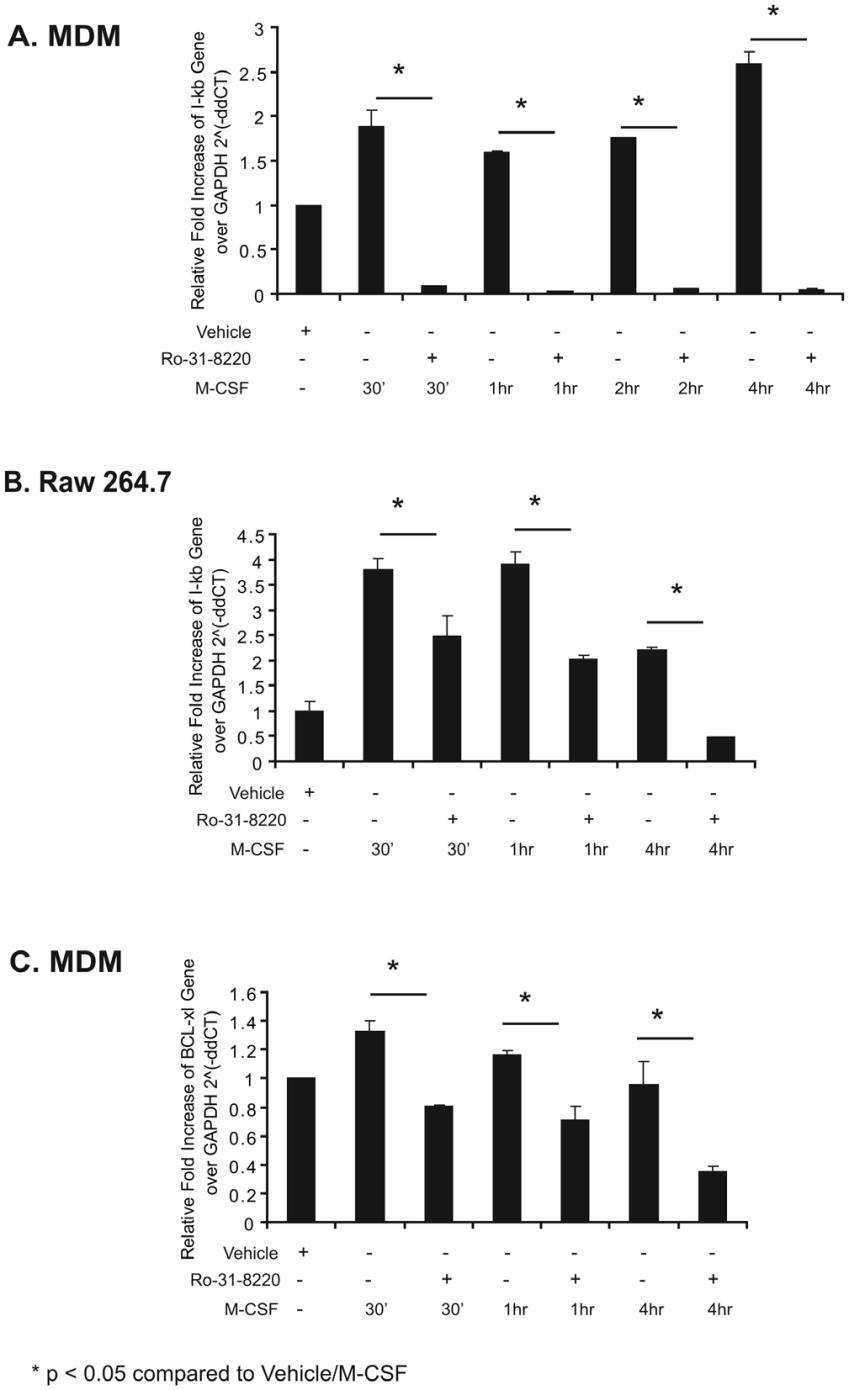
Inhibition of M-CSF-induced PKC reduces NF-κB-regulated genes in both MDMs and RAW 264.7 cells. MDMs (**A and C**) and RAW 264.7 (**B**) cells were pretreated with Ro-31-8220 or solvent control DMSO for 30 minutes prior to M-CSF stimulation for the indicated times. Total RNA was isolated and converted to cDNA. Real-time RT PCR was performed using primers for IκBα, BCL-xl or GAPDH. Data are expressed as relative fold increase of IκBα or BCL-xl gene expression upon treatment over non-stimulated cells. Data represent the mean ± S.E.M for three independent experiments.

NF-κB induces a number of downstream genes, including the IκB family. Among the IκB molecules, IκBα is highly induced by NF-κB activation [40]. Having shown that PKC regulated NF-κB activity in M-CSF-stimulated MDMs, we next determined whether inhibition of PKC activity decreased expression of NF-κB-regulated genes. We treated both MDMs and RAW 264.7 cells with the PKC inhibitor Ro-31-8220 for 30 minutes and then stimulated with M-CSF. IκBα gene was measured by qRT-PCR. As shown in Figures 6A and 6B, M-CSF enhanced IκBα gene expression and PKC inhibition by Ro-31-8220 decreased IκBα gene expression in both MDMs and RAW 264.7 cells (p<0.01), demonstrating that PKC affected NF-κB-regulated gene expression in macrophages.

To further define the role of PKC in mediating human MDM survival in response to M-CSF, we examined the expression of the anti-apoptotic gene BCL-xL, which is also regulated by NF-κB. As shown in Figure 6C, Ro-31-8220 reduced M-CSF-stimulated BCL-xL expression compared to cells treated with M-CSF and the vehicle control DMSO (p<0.05).

### Identification of PKCα as the Upstream Activator of NF-κB in Myeloid Cells

**Figure 7:**
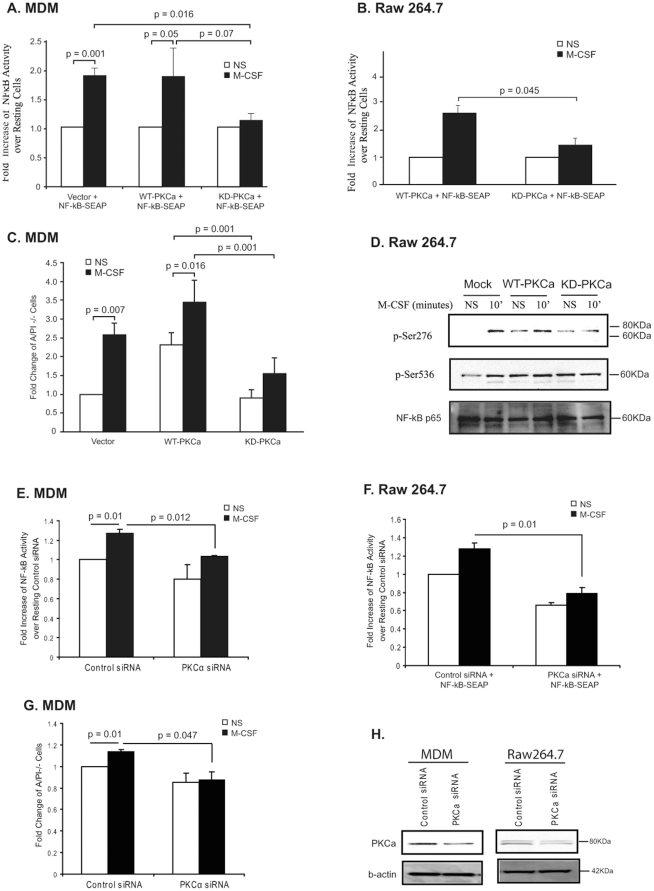
PKCα regulates phosphorylation of NF-κB p65 at Ser276. (**A**) MDM or (**B**) RAW 264.7 cells were transiently transfected with pNF-κB-SEAP along with either WT-PKCα or the kinase-deficient (KD)-PKCα construct at a 1∶5 ratio. Cells were serum starved and stimulated with M-CSF and then SEAP secretion in the medium was measured. Data is from of three independent experiments. The p-value of cells transfected with KD compared to those transfected with WT was 0.05. (**C**) MDMs were removed from the plate using accutase and apoptosis of MDMs was measured by flow cytometry using Annexin V-FITC and propidium iodine (PI). (**D**) Whole cell lysates from the transfected RAW 264.7 cells were subjected to Western blot analysis with phospho-Ser276 or phospho-Ser536 NF-κB p65 antibodies. Blots were immunoblotted with PKCα to determine equal protein expression for the PKCα constructs. β-actin served as a loading control. Shown is a representative blot from three independent experiments. (**E**) MDM or (**F**) RAW 264.7 cells were transiently transfected with a pNF-κB-SEAP along with either 100 nM PKCα siRNA or control siRNA for 20-24 hours. Cells were serum starved for 2-4 hours and stimulated with 100 ng/ml M-CSF for 6 hours for MDM or RAW 264.7 for 2 hours and then SEAP secretion in the medium was measured. Shown is data of three independent experiments. (**G**) MDMs were removed from the plate using accutase and apoptosis of MDMs was measured by Annexin V-FITC and propidium iodine (PI) staining and analyzed by flow cytometry. (**H**) Whole cell lysates from the transfected MDM and RAW 264.7 cells were subjected to Western blot analysis with PKCα antibody. β-actin served as a loading control. Shown is a representative blot from at least three independent experiments.

Even though the PKC family consists of 10 members, finding that PKCα/β inhibitors and intracellular calcium inhibitors reduced M-CSF-induced NF-κB activity, suggested PKCα was involved in NF-κB activation after M-CSF treatment. To confirm the role of PKCα in NF-κB activation in macrophages, constructs for either wildtype (WT)-PKCα or kinase-deficient (KD)-PKCα was co-transfected with the pNF-κB-SEAP reporter gene and SEAP secretion was measured. As shown in Figure 7A, MDMs co-transfected with pNF-κB-SEAP and WT-PKCα had a 1.8-fold increase in NF-κB transcriptional activity after M-CSF activation compared with NS cells (p = 0.05), similar to M-CSF-treated cells expressing only pNF-κB-SEAP. Transfecting human macrophages with the KD-PKCα construct significantly reduced M-CSF-induced NF-κB activity compared to WT-PKCα transfected cells (p = 0.016). Similarly, RAW 264.7 cells transfected with WT-PKCα had 2.5-fold more NF-κB transcriptional activity after M-CSF activation compared to unstimulated RAW 264.7 cells (NS) transfected with WT-PKCα (Figure 7B). Expression of the KD-PKCα construct into RAW 264.7 cells reduced M-CSF-induced NF-κB activity to 1.5-fold (p = 0.045) compared to cells transfected with WT PKCα.

Cell survival was also examined in MDMs expressing either WT-PKCα or KD-PKCα constructs by Annexin V-FITC and PI staining. As expected, M-CSF increased MDM survival as measured by the percent of Annexin V/PI negative cells. Similarly, expression of WT-PKCα protected cells from apoptosis. In contrast, expression of KD-PKCα decreased M-CSF-induced cell survival (p<0.01) (Figure 7C).

Next, we examined the effect of expressing the PKCα constructs on NF-κB phosphorylation. As shown in Figure 7D, expression of KD-PKCα in RAW 264.7 cells did not affect the constitutive phosphorylation at Ser536 of NF-κB p65, but attenuated the phosphorylation at Ser276. Expression of WT-PKCα did not effect the phosphorylation of either residue with or without M-CSF stimulation. These observations demonstrate that PKCα is important in M-CSF-regulated cell survival and NF-κB activation and likely regulated through phosphorylation of Ser276 of NF-κB p65.

To further validate the impact that PKCα played in M-CSF-induced NF-κB transcriptional activity, we next employed PKCα siRNA treatment of MDM or RAW cells. A pool of specific PKCα siRNA were transfected into MDM or Raw 264.7 cells in the presence or absence or M-CSF. Reducing native PKCα expression decreased M-CSF-induced NF-κB transcriptional activity in both MDM (Figure 7E) (p = 0.012) and Raw 264.7 cells (Figure 7F) (p = 0.01). We also examined cell survival of the MDMs by Annexin V-FITC and PI staining after PKCα siRNA transfection. As shown in Figure 7G, M-CSF-induced MDM survival was reduced in the cells transfected with PKCα siRNA compared with cells transfected with control siRNA (p = 0.047). In Figure 7H, we confirmed that PKCα siRNA transfection decreased PKCα protein expression in both MDM and Raw 264.7 cells. Our results indicated that PKCα regulated NF-κB activation and M-CSF-regulated cell survival.

### PKC is Essential in Regulating NF-κB Activity

**Figure 8:**
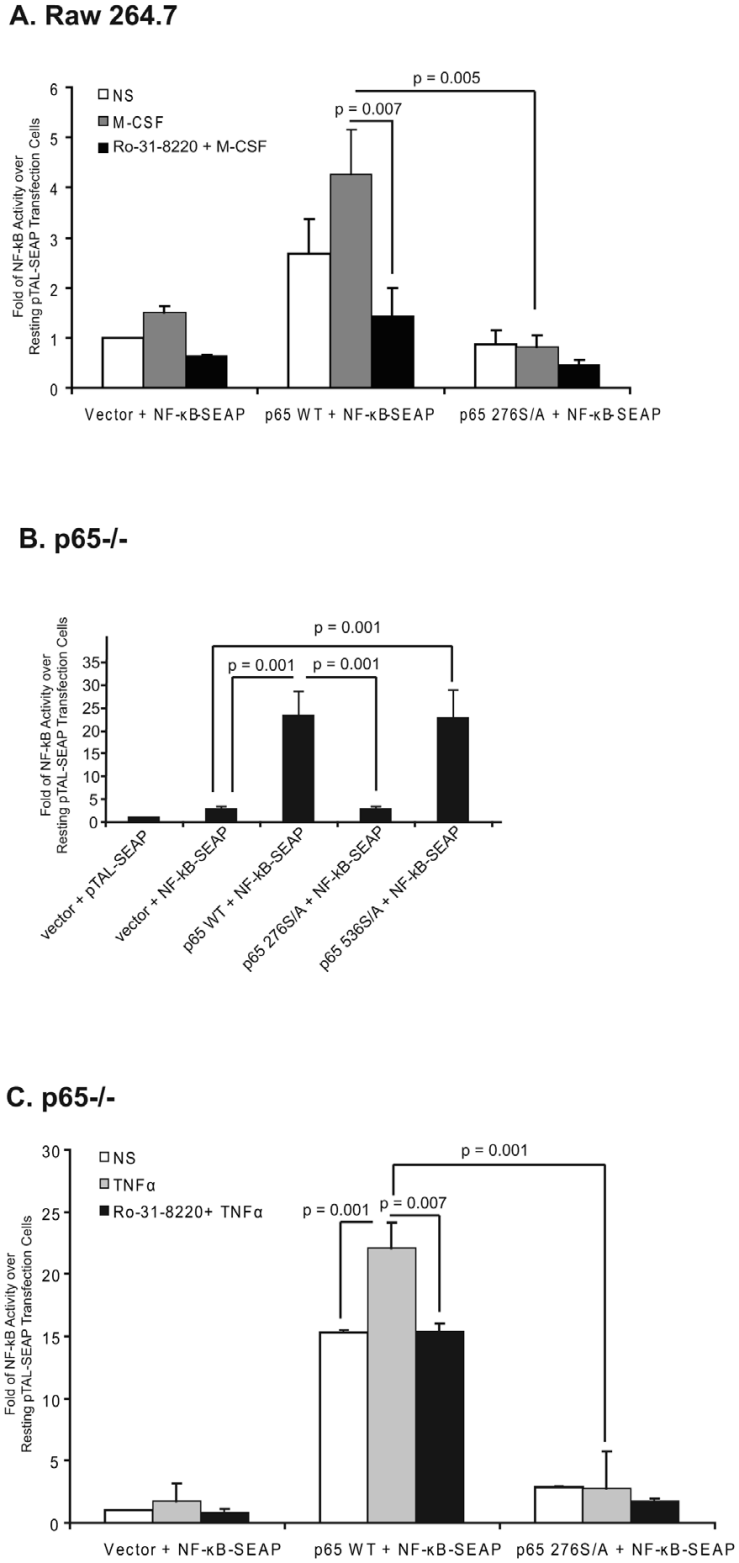
NF-κB p65 Ser276 is essential in regulating NF-κB activity. (**A**) Raw 264.7 cell line was transiently transfected with pNF-κB-SEAP along with empty vector or plasmid encoding either NF-κB p65 WT or NF-κB p65 276S/A. The cells were transfected for 18-24 hours, serum starved for 4 hours, and then incubated with 10 µM of Ro-31-8220 for 30 minutes prior to treatment with 100 ng/ml of M-CSF for 2 hours and SEAP secretion in the medium was measured. (**B**) NF-κB p65−/− cell line was transiently transfected with pNF-κB-SEAP along with empty vector or plasmid encoding either NF-κB p65 WT, NF-κB p65 276S/A, or NF-κB p65 536S/A. The cells were cultured for 24 hours and then serum starved for 4 hours. Cells were then incubated in fresh DMEM medium for 2 hours and SEAP secretion in the medium was measured. The results shown are fold change over empty vector + pTAL-SEAP. (**C**) NF-κB p65−/− cell line was transiently transfected with pNF-κB-SEAP with either empty vector or plasmid encoding either NF-κB p65 WT or NF-κB p65 276S/A. The cells were transfected for 24 hours and serum starved for 4 hours, and then incubated with 10 µM of Ro-31-8220 for 30 minutes prior to treatment with 10 ng/ml of TNFα. The supernatant were collected after 2 hours of treatment and SEAP secretion in the medium was measured. The results shown are the fold change over empty vector + pNF-κB-SEAP. Data shown are mean ± S.E.M for at least three independent experiments performed in duplicate.

M-CSF-dependent PKC activity facilitated NF-κB p65 phosphorylation at Ser276 but not Ser536. To confirm the role of PKC and p65 Ser276 phosphorylation on NF-κB activity, we co-transfected the NF-κB-SEAP construct with either WT NF-κB p65 or NF-κB p65 276S/A constructs in Raw 264.7 cells, then treated cells with the PKC inhibitor Ro-31-8220 in the absence or presences of M-CSF. As expected in cells transfected with vector control, inhibiting PKC reduced M-CSF-stimulated NF-κB activity compared to cells treated with M-CSF and the vehicle DMSO (p = 0.001) (Figure 8A). In contrast, expressing WT NF-κB p65 (p65 WT) increased NF-κB activity, while the general PKC inhibitor Ro-31-8220 decreased this NF-κB activity (p = 0.001). Notably, the introduction of NF-κB p65 276 S/A construct significantly reduced NF-κB activity compared with the WT NF-κB p65 construct (p = 0.005) and M-CSF treatment was unable to overcome this inhibition.

Furthermore, we co-transfected the NF-κB-SEAP construct along with either the WT NF-κB p65, NF-κB p65 276S/A or NF-κB p65 536S/A constructs into a NF-κB p65−/− murine fibroblast cell line and measured NF-κB activity. As predicted, expression of WT NF-κB p65 (p65 WT) in NF-κB p65−/− cell line constitutively activated NF-κB compared to cells transfected with vector control without any stimulation (Figure 8B). In comparison, transfecting the NF-κB p65 276S/A construct reduced NF-κB activity by 5-fold (p = 0.006, WT NF-κB p65 vs. NF-κB p65 276S/A), while expressing the NF-κB p65 536S/A construct increased NF-κB activity in the p65−/− cells to levels similar to WT NF-κB p65 transfected cells.

Since M-CSF-induced PKC activation regulated NF-κB activity via Ser276 residue of NF-κB p65 in primary human MDMs and RAW 264.7 cells, we next examined if this occurred in NF-κB p65−/− fibroblasts in response to a native stimulus for these cells, TNFα. NF-κB activity was measured in the WT NF-κB p65 or NF-κB p65 276S/A transfected cells treated with TNFα in the absence or presence of Ro-31-8220 (Figure 8C). As expected, TNFα increased NF-κB activity in NF-κB p65−/− cells expressing WT p65 and (p = 0.001) PKC inhibitors decreased NF-κB activity to the non-stimulated (NS) level (p = 0.007). Introducing NF-κB p65 276 S/A constructs significantly reduced NF-κB activity compared with the WT NF-κB p65 construct (p = 0.001). Notably, TNFα failed to increase NF-κB activity in the NF-κB p65−/− cells expressing NF-κB p65 276S/A constructs. These observations are similar to macrophages overexpressing the NF-κB p65 276S/A (Figure 8A). Our data demonstrate that Ser276 of NF-κB p65 is essential in regulating NF-κB activity and suggests that PKC regulates NF-κB activity by modulating the phosphorylation of NF-κB p65 at Ser276 residue.

## Discussion

**Figure 9:**
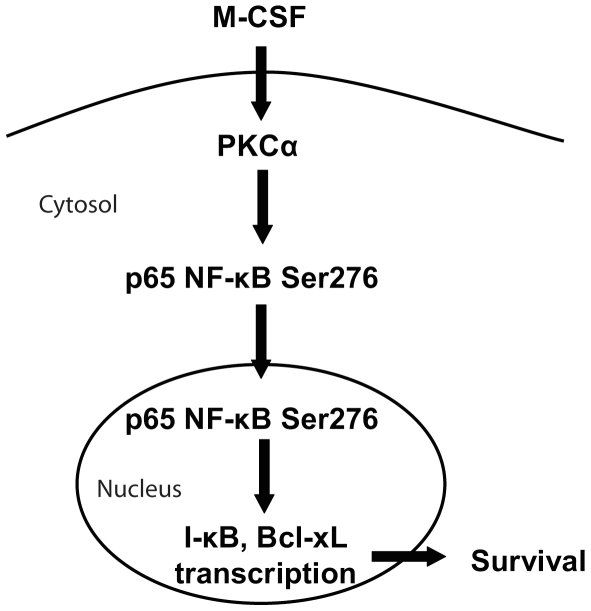
Proposed model for M-CSF-induced monocyte survival via PKC regulation by activating NF-κB p65 phosphorylation at Ser276.

NF-κB transcriptional activity is important in regulating intracellular signaling, stress response, proliferation, survival, differentiation and inflammation [8]. To regulate gene transcription, NF-κB p50/p65 heterodimers translocate to the nucleus from the cytoplasm, however, their translocation is not sufficient to activate gene transcription. Post-translational modification of NF-κB heterodimers such as phosphorylation or acetylation also contributes to its transcriptional activity [41], [42], [43]. Because NF-κB constitutively translocates to the nucleus in monocytic lineage [13] and activates gene transcription and survival in murine macrophages [11], we sought to determine how M-CSF affected NF-κB transcriptional activity in human MDMs and whether this activation regulated their survival. The present study demonstrated that M-CSF stimulated PKCα kinase upstream of NF-κB transcriptional activity in primary human MDMs and RAW 264.7 cells. We showed inhibition of conventional PKCs by PKC inhibitors, kinase deficient PKCα or PKCα siRNA blocked M-CSF-induced cell survival and NF-κB-regulated gene expression. This block correlated with a reduction in M-CSF-induced phosphorylation of NF-κB p65 at Ser276. Consistent with these findings the dominant negative PKCα constructs also inhibited NF-κB p65 phosphorylation at Ser276 but not at Ser536 resulting in reduced NF-κB transcriptional activation and M-CSF-induced MDM survival. A simplified proposed cartoon model in Figure 9 demonstrates that M-CSF induced monocytes survival is regulated by activating NF-κB p65 phosphorylation at Ser276 via PKC. Finally, in a NF-κB p65−/− fibroblast cell line, we confirmed that the Ser276 residue of NF-κB p65 is important and essential for PKC modulation of NF-κB activity. More compelling is that we observed a similar regulation between two different cell lineages in our study.

Post-translational modification of NF-κB p65 regulates its activating or repressing effects on gene expression. Among the seven reported putative NF-κB p65 phosphorylation sites, five increase nuclear translocation, DNA binding and NF-κB transcriptional activity [17]. Similar to our finding, Zhong et al. found that in response to LPS, NF-κB p65 is phosphorylated at the highly conserved Ser276 residue by the catalytic subunit of PKA [16]. In addition to PKA, Ser276 can be phosphorylated by MSK1 in the nucleus upon TNFα treatment [18]. Phosphorylation of NF-κB p65 at Ser536 occurs in response to many inflammatory stimuli as well as kinases, IKKα, IKKβ and RSK1 [17], [18], [19], [20], [21]. Notably, in addition to phosphorylating Ser276 of NF-κB, our study revealed that M-CSF also modulated the phosphorylation of the NF-κB p65 subunit at Ser536, however, PKC inhibitors or kinase deficient PKCα constructs did not affect this phosphorylation event. Moreover, mutant constructs containing a point mutation at Ser536 of NF-κB p65 did not reduce NF-κB activity in cells lacking endogenous NF-κB p65, while the NF-κB p65 276S/A construct did reduce NF-κB activity in these cells. Therefore, specific post-translational modification of NF-κB p65 is likely to regulate transcriptional activation in response to specific stimuli [15], [43].

It is notable that M-CSF activated NF-κB p65 Ser276 phosphorylation and transcriptional activation in a PKCα-dependent manner. PKCα is a member of the conventional PKC family of protein kinases that are critical for cell growth, differentiation and cell death. PKCα primarily modulates anti-apoptotic and proliferation signals following cytokine treatment in various cell types [42]. Expression of PKCα attenuates apoptosis in many different cell types, while PKCα inhibition generally potentiates apoptosis [42]. In our studies using conventional PKC inhibitors and dominant negative PKCα constructs, PKCα was critical in M-CSF-induced macrophage survival by inducing Ser276 phosphorylation of NF-κB p65 in both primary human MDMs and Raw 264.7 cells. We also demonstrated PKC inhibition downregulated NF-κB-induction of the anti-apoptotic gene BCL-xL. Consistent with our finding, others reported NF-κB regulated the gene expression of many anti-apoptotic proteins including other BCL-2 family members and inhibitor of apoptosis (IAP) proteins which are regulators of apoptosis and function upstream of caspases [44]. It has been shown that phosphorylation of p65 at Ser276 prevents its degradation by ubiquitin-mediated proteolysis and promotes cell survival in HeLa cells [24]. In addition to BCL-xL expression as examined in this study, we are evaluating the possibility that PKCα-regulated NF-κB activity upregulates additional anti-apoptotic genes in an M-CSF-dependent fashion to modulate cell survival.

The use of inhibitors and siRNA in our study has not ruled out that other conventional PKCs might also be important in M-CSF-induced NF-κB activation and macrophage survival. In contrast to the actions of PKCα activation of other conventional PKC isoforms, like PKCβI/βII, appear necessary for macrophage apoptosis. The PKCβI/βII isoforms are expressed in apoptotic U937 myelomonocytic cells [45]. The human myeloid cell line HL-525 which is devoid of endogenous PKCβII is resistant to TNFα-induced apoptosis [46]. Re-expression of PKCβII in HL-525 cells restores their susceptibility to TNFα-induced apoptosis implying that PKCβII is pro-apoptotic and may be required to induce macrophage apoptosis.

Recent studies demonstrated that NF-κB transcriptional activity can be regulated by phosphorylation of the NF-κB p65 subunit in the absence of IκBα degradation [47]. By this pathway, phosphorylation of NF-κB regulates its interaction with other components of the basal transcriptional machinery without affecting its capability to bind DNA [21]. Since NF-κB can bind the promoters of numerous gene targets, post-translational modification of NF-κB p65 may affect its interaction with other proteins, refining the expression of gene targets. We did not find that PKC was involved in IκBα degradation or NF-κB p65 nuclear translocation induced by M-CSF, but that M-CSF-induced phosphorylation of NF-κB p65 at Ser276 was dramatically reduced by the conventional PKC inhibitor and kinase deficient PKCα and by siRNA towards PKCα. Current studies are underway to delineate the protein complexes formed by activated NF-κB and other transcriptional co-activators in response to M-CSF and PKC activation. Recently studies indicated that the non-canonical pathway was activated during human monocyte-macrophage differentiation [48]. During this process, expression of IKKα among other proteins, is elevated leading to increased p52 NF-κB (relB) expression through partial proteolytic degradation of the p100 NF-κB protein. Thus, it is possible that M-CSF regulates monocyte survival through both the canonical and non-canonical NF-κB pathways, which will be a focus in future research.

We previously showed that M-CSF reduced caspase-3 and -9 activity and prevented monocyte apoptosis [3]. This study reveals that treatment of monocytes with conventional PKC inhibitors, or overexpression of kinase-deficient PKCα blocked M-CSF-induced cell survival. Our data revealed that conventional PKCs are upstream of NF-κB activation in response to M-CSF and mediate post-translational activation of NF-κB p65 by phosphorylating Ser276 to regulate gene transcription and cell survival. Thus, our observation may provide insight in potential therapeutic targets for inflammatory diseases.

## Materials and Methods

### Materials

Recombinant human M-CSF was purchased from R&D Systems (Minneapolis, MN). Ro-31-8220, Gö-6976, Rotterin, and BAPTA/AM were obtained from Calbiochem (San Diego, CA). Low endotoxin RPMI and X-vivo serum free medium were obtained from Lonza Walkersville Inc (Walkersville, MD). Fetal bovine serum (FBS, certified <0.06 endotoxin units/ml endotoxin levels) was purchased from Atlanta Biological (Lawrenceville, GA). All other cell culture reagents were obtained through Invitrogen (Carlsbad, CA). PKCα NF-κB p65, Lamin B, β-actin, and GAPDH antibodies, anti-mouse and rabbit IgG-HRP conjugated antibodies, and PKCα siRNA were obtained from Santa Cruz Biotechnology (Santa Cruz, CA). Phospho-NFκB p65 (Ser276 or Ser536) antibodies were purchased from Cell Signaling Technology (Danvers, MA). DNA constructs pTAL-SEAP and pNF-κB-SEAP were obtained from Clontech (Mountian View, CA). Expression vectors containing CMV-p65 WT, CMV-p65 276S/A and CMV-p65 536S/A were cloned in pBa5e Puro vector or pFLAG-CMV-2 vector as described previously [49]. Kinase deficient CMV-PKCα (KD-PKCα) or wild type pCMV-PKCα (WT-PKCα) were generous gifts from Alexandra C. Newton (University of California, San Diego, CA). IκBα (NFKBIA) and GAPDH primers for real-time RT-PCR were obtained from SABiosciences (Frederick, MD). A pool of mouse or human PKCα specific siRNA were purchased from Santa Cruz Biotechnology (Santa cruz, CA).

### Cell Culture

The murine macrophage cell line RAW 264.7 was purchased from ATCC (Manassas, VA). RAW 264.7 cells were maintained in RPMI supplemented with 5% FBS and antibiotic-antimycotic (1000 U/ml penicillin, 1000 µg/ml streptomycin sulfate, and 250 ng/ml amphotericin B) at 37°C. Mouse embryonic fibroblasts (MEFs) cell line lacking specific NFκB signaling subunits NF-κB p65 (p65−/− cell line) [50] were cultured in DMEM medium supplemented with 10% FBS and antibiotic-antimycotic solution.

### Purification of Peripheral Blood Monocytes and Monocyte-Derived Macrophages (MDMs)

Monocytes were isolated from source leukocyte packs obtained from the American Red Cross as described previously [51]. Monocytes used in real time PCR experiments and transfection experiments were purified by positive selection using CD14 Monocyte Isolation Kit from Miltenyi Biotech (Auburn, CA) (>90% pure). In some experiments, monocytes were isolated by clumping method (70% pure). To obtain monocyte-derived macrophages (MDMs), monocytes were cultured in RPMI-1640 medium containing 10% FBS, and 10 µg/ml polymyxin B and 20 ng/ml M-CSF.

### Electrophoresis of the Mobility Shift Analysis (EMSA)

Nuclear extracts were isolated from MDMs by using a nuclear extraction kit according to the manufacturer's instruction from Active Motif (Carlsbad, CA). Briefly, MDMs were lysed in hypotonic lysis buffer (20 mM Hepes, pH 7.5; 5 mM NaF, 10 µM Na_2_MoO_4_ 0.5% NP-40 and 0.1 mM EDTA), and then nuclei were resuspended in lysis buffer supplemented with 0.5 mM DTT and 0.2 mM PMSF. The NF-κB consensus oligonucleotides (sense: AGTTGAGGGGACTTTCCCAGGC; antisense: GCCTGGGAAAGTCCCCTCAACT) labeled with ^32^P by T4 polynucleokinase (Promega, Madison, WI) were incubated with nuclear extracts in binding buffer (10 mM Tris pH 7.6, 1 mM DTT, 0.5 mM EDTA, 2 µg polydI-dC and 10% Glycerol) at 30°C for 30 minutes. The free DNA and DNA-protein mixtures were resolved in 5% native polyacrylamide gels in 0.5× TBE buffer (45 mM Tris, 45 mM boric acid and 1 mM EDTA, pH 8.3) by electrophoresis. The gel was dried and subjected to autoradiography analysis.

### Transient Transfection of RAW 264.7 Cells

RAW 264.7 cells were seeded in 12-well plates in RPMI medium containing 5% FBS one day prior to transfection, Secreted alkaline phosphatase (SEAP) reporter plasmids pTAL-SEAP or pNF-κB-SEAP were transfected into cells using Qiagene Effectene or Attractene transfection kit (Qiagen, Valencia, CA) according to the manufacturer's protocol. For co-transfection studies, PKCα or NF-κB p65 constructs were mixed at a ratio of 5∶1 with the reporter plasmid. For siRNA transfection, 100 nM of PKCα siRNA or control siRNA were used. Cells were incubated with the transfection reagents for 16–24 hours, washed and starved in RPMI without FBS for 4 hours. Samples were then stimulated with 100 ng/ml M-CSF for 4 hours at which point the medium was collected for the SEAP analysis. For inhibition studies, cells were pre-incubated with inhibitors for 30 minutes before M-CSF treatment. On average, we realized 30–40% transfection efficiency as confirmed by GFP expression in Raw 264.7 cells.

### Transient Transfection of Primary Human Monocytes

Monocytes were transfected using the human monocyte Amaxa nucleofector kit (Lonza Walkersville Inc, Walkersville, MD) as previously described [51]. Briefly, monocytes were resuspended in Amaxa Nucleofactor solution at a density of 20×10^6^ cells/ml, and 2 µg of total plasmid DNA 100 nM of PKCα siRNA or control siRNA was added and transfected using program Amaxa Y-01. For co-transfection studies, PKCα or NF-κB p65 constructs were mixed at a ratio of 5∶1 with the reporter plasmid. Immediately after transfection, cells were washed with 1 ml of RPMI medium containing 2 mM glutamine and 10% FBS or serum free LGM medium (Lonza Walkersville Inc.) then plated at 4×10^5^ cells/well in 24-well plates. The original medium was replaced by serum free LGM medium without or with M-CSF (100 ng/ml) one hour later. The next day, cell-free supernatants were collected for the SEAP analysis. Cells were stained with Annexin V/propidium iodide (PI). For the inhibition studies, cells were pre-incubated with inhibitor for 30 minutes in X-vivo medium prior to the addition of M-CSF.

### Secreted Alkaline Phosphatase (SEAP) Analysis

Secreted alkaline phosphatase activity was analyzed by GreatEscape SEAP kit according to the manufacturer's instruction. Briefly, medium was diluted in the dilution buffer and heated to 65°C to inactivate endogenous phosphatases then incubated with the substrate for 30 minutes. The chemiluminence signal was recorded using a luminometer.

### Annexin V/PI Apoptosis Assay

Cell apoptosis assay was performed as described previously [51] using the Annexin V–FITC apoptosis detection kit (BD PharMingen, San Diego, CA). Briefly, human monocytes (5×10^6^) were incubated with inhibitors for 30 minutes in X-vivo medium and restimulated with 100 ng/ml M-CSF overnight. The cells were removed from the culture dish using Accutase (eBioscience, San Diego, CA) and stained with Annexin V–FITC and PI and analyzed by flow cytometry (FACSCalibur; BD PharMingen). Annexin V-FITC and PI double negative cells were considered non-apoptotic cells for statistical analysis.

### Western Blot Analysis

Cells were washed with PBS and resuspended in cell lysis buffer. (Cell Signaling Technology) and incubated for 10 minutes on ice then centrifuged to remove the insoluble fraction. The protein concentration was determined by the BCA protein assay (Bio-Rad, Hercules, CA). Cell lysates were separated by SDS-PAGE on 10% polyacrylamide gels and then transferred onto nitrocellulose membranes and subjected to Western blotting. The immunoblotted proteins were detected by ECL reagent (GE Healthcare Bio-Sciences Corp., Piscataway, NJ).

### Analysis of PKCα Kinase Activity

Human MDMs were starved for 2 hours before stimulation with M-CSF (0–60 minutes), then washed with cold PBS and removed from the plates. The cells were resuspended in lysis buffer (20 mM Tris, 5 mM MgCl_2_, 1 mM EGTA, 20 mM β-glycerol phosphate, 1 mM PMSF 2 µg/ml aprotinin and 2 µg/ml leupeptin, pH 7.5), sonicated and centrifuged to obtain whole cell lysates, which were then immunoprecipitated with anti-PKCα antibody and protein G-agarose (Invitrogen). Immune complexes were washed twice, and PKCα activity was analyzed by non-radioactive Peptag assay kit (Promega). Briefly, kinase buffer, activator, peptide protection solution and Peptag peptide were incubated with the immune complexes at 30°C for 30 minutes. Reactions were stopped by boiling for 10 minutes and samples were separated on 0.8% agarose gel. Phosphorylated peptide migrated toward the anode (+), while non-phosphorylated peptide migrated toward the cathode (−). Fluorescein-tagged peptides were visualized by UV light.

### RNA Isolation and Quantitative Real-time PCR

MDMs or RAW 264.7 cells were serum-starved overnight or for 2 hours, respectively, prior to incubating with inhibitors for 30 minutes. The cells were restimulated with 100 ng/ml M-CSF and total mRNA was extracted from cells with Trizol (Invitrogen) and 1–2 µg of total RNA was used to synthesized cDNA using SuperScript III (Invitrogen). Quantitative real-time (qRT)-PCR was performed using SYBR Green Master Mix (Applied BioSystems, Carlsbad, CA). The reactions were performed using an ABI PRIZM 7700 machine with software Sequence Detector version 1.7 (Applied Biosystems). The target gene values were normalized to the values of GAPDH as a housekeeping gene and expressed as relative fold increase 2^(−ΔΔCt)^ over the non-stimulated samples (NS).

### Statistical Analysis

Statistical comparisons were performed using analysis of variance testing (Minitab software, State Park, PA or SPSS16 software, SPSS Inc. Chicago, IL). Statistical significance was defined as p≤0.05.

### Ethics Statement

All research involving human blood (Buffy coat) were ordered from American Red cross and have been approved by the Ohio State University review board (ARC IRB Protocol #2001-26).

## Supporting Information

Figure S1 **Expression of c-Rel does not enhance M-CSF-induced NF-κB transcriptional activity in macrophages.** RAW 264.7 cells were transiently transfected with pTAL-SEAP, pNF-κB-SEAP or pNF-κB-SEAP + HA-cRel constructs. Cells were serum starved for 4 hours prior to the stimulation with mouse recombinant M-CSF (100 ng/ml) for 2 hours. Culture media was collected to measure SEAP production. Data are expressed as fold increase of SEAP activity over that in pTAL-SEAP transfected resting cells. The graph represents mean ± S.E.M for two independent experiments done in triplicates.
(TIF)
