# Supplementary figures and images for: M-CSF Induces Monocyte Survival by Activating NF-κB p65 Phosphorylation at Ser276 via Protein Kinase C
Source: PLoS One. 2011 Dec 22;6(12):e28081. doi: 10.1371/journal.pone.0028081 (PMC3245220; doi:10.1371/journal.pone.0028081)

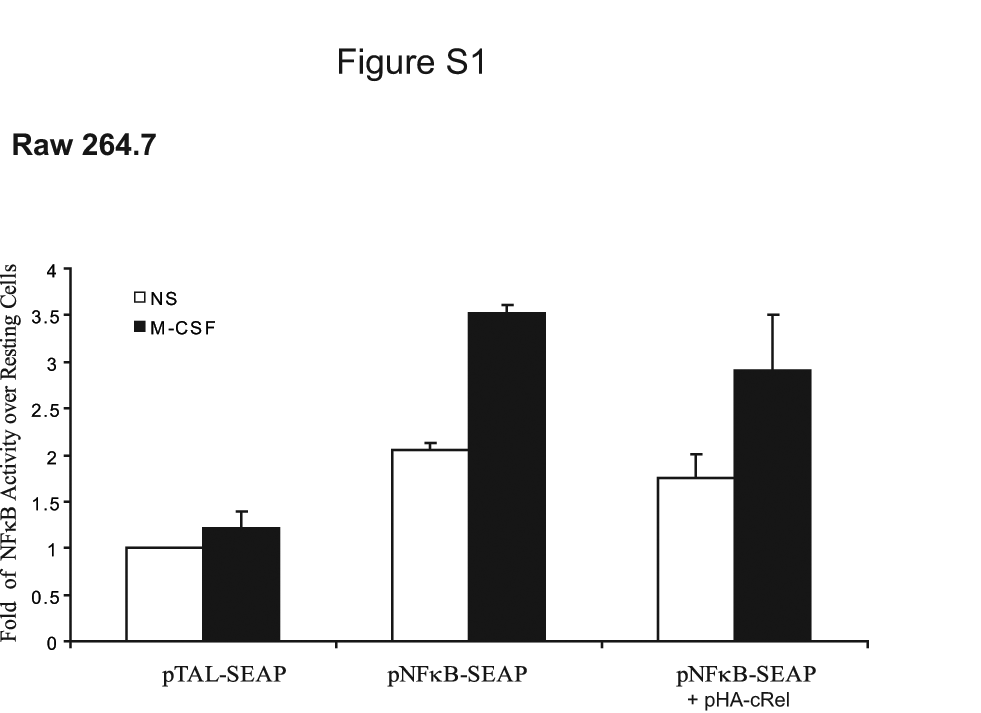

Supplement: Figure S1 — Expression of c-Rel does not enhance M-CSF-induced NF-κB transcriptional activity in macrophages. RAW 264.7 cells were transiently transfected with pTAL-SEAP, pNF-κB-SEAP or pNF-κB-SEAP + HA-cRel constructs. Cells were serum starved for 4 hours prior to the stimulation with mouse recombinant M-CSF (100 ng/ml) for 2 hours. Culture media was collected to measure SEAP production. Data are expressed as fold increase of SEAP activity over that in pTAL-SEAP transfected resting cells. The graph represents mean ± S.E.M for two independent experiments done in triplicates. (TIF) [file pone.0028081.s001.tif]
